# Risk Factors and Prognosis of New-Onset Atrial Fibrillation in Sepsis: A Nationwide Electronic Health Record Study

**DOI:** 10.1016/j.jacadv.2025.101681

**Published:** 2025-04-23

**Authors:** Yuxin Huo, Hiroyuki Yoshimura, Arturo Gonzalez-Izquierdo, Gregory Y.H. Lip, Floriaan Schmidt, Rui Providencia

**Affiliations:** a Institute of Health Informatics, University College London, London, United Kingdom; b Centre for Health Data Science, Institute of Applied Health Research, University of Birmingham, Birmingham, United Kingdom; c Liverpool Centre for Cardiovascular Science at University of Liverpool, Liverpool John Moores University and Liverpool Heart & Chest Hospital, Liverpool, United Kingdom; d Danish Center for Health Services Research, Department of Clinical Medicine, Aalborg University, Aalborg, Denmark; e Department of Cardiology, Amsterdam University Medical Centres, Amsterdam, The Netherlands; f Institute of Cardiovascular Science, University College London, London, United Kingdom; g Division of Heart and Lungs, University Medical Center Utrecht, Utrecht, Netherlands; h Barts Heart Centre, St Bartholomew's Hospital, London, United Kingdom

**Keywords:** arrhythmia, incidence, infection, prognosis, risk factors

## Abstract

**Background:**

Atrial fibrillation (AF) may occur in patients with sepsis and is associated with a worse prognosis. To date, no UK nationwide studies have investigated the risks and impact of AF and sepsis.

**Objectives:**

The authors aimed to: 1) identify risk factors contributing to the development of new-onset AF in patients with sepsis; and 2) assess the impact of new-onset AF on in-hospital and long-term outcomes.

**Methods:**

Utilizing linked UK-electronic health records of 5.6 million people between 1998 and 2016, we analyzed risk factors for new-onset AF in the setting of sepsis and assessed duration of hospitalization, rate of septic shock, 7- and 30-day in-hospital mortality, postdischarge mortality, and stroke. Cox proportional hazards models were used to assess postdischarge outcomes, and adjustment for behavioral and demographic variables, and comorbid conditions was performed. Fine-Gray analyses were used to account for competing risks.

**Results:**

We identified 7,691 patients hospitalized for sepsis and new-onset AF, 24,506 patients with sepsis who did not develop new-onset AF, and 95,287 patients hospitalized for new-onset AF who did not have sepsis. Age, sex, ethnicity, socioeconomic deprivation, smoking, chronic obstructive pulmonary disease, heart failure, ischemic heart disease, valvular heart disease, and hypertension were significantly associated with new-onset AF. Compared to sepsis patients without AF, those with new-onset AF during sepsis had longer duration of hospitalization, higher risk of developing septic shock, and higher in-hospital mortality. Patients with sepsis and new-onset AF had a higher rate of stroke (adjusted HR: 1.18; 95% CI: 1.08-1.30), heart failure, myocardial infarction, and mortality postdischarge (adjusted HR: 1.07; 95% CI: 1.03-1.12) than those with sepsis without AF.

**Conclusions:**

AF during sepsis is common and is not an innocent finding. Active monitoring should be pursued as AF has important short- and long-term prognostic implications.

Atrial fibrillation (AF) is the most prevalent sustained cardiac arrhythmia and is a significant cause of vascular-related disease and mortality.^1^ Although the focus of AF management has been on cardiovascular risk factors and complications such as hypertension, stroke, and heart failure, recent evidence suggests that noncardiovascular comorbidities are also common,^2^ with international guidelines recommending holistic or integrated care management as part of the Atrial fibrillation Better Care pathway for the management of these patients.^3^

Sepsis is a potentially life-threatening condition of organ failure resulting from an imbalanced response of the host to infection^4^ and occurs in nearly 49 million people annually worldwide.^5^ For example, An et al^6^ reported that sepsis was among the 3 main causes of AF-related mortality (ie, malignancy, sepsis, and heart failure). New-onset AF has been observed in 5% to 25% of patients with sepsis^7^^,^^8^ and is associated with higher risk of hemodynamic instability, more prolonged intensive care unit stay, and subsequently having further AF episodes, or AF-related complications.^7^^,^^9^, ^10^, ^11^

Although studies have previously documented the association between sepsis and AF, the specific underlying risk factors for new-onset AF during sepsis and the long-term prognosis postdischarge remain unclear. Given that sepsis and AF are both highly prevalent medical conditions with high clinical risk, it is critical to understand their inter-relationships and prognostic implications. No European nationwide studies have as yet investigated this matter. Furthermore, the European Society of Cardiology, American College of Cardiology, American Heart Association AF guidelines, and Surviving Sepsis Campaign international guidelines for management of sepsis and septic shock all fail to provide specific management recommendations for patients with sepsis and new-onset AF, showing that raising awareness of the medical community to this particular group of patients is required.

Using a UK nationwide data set, we aimed to: 1) identify risk factors that contribute to the development of new-onset AF in patients with sepsis; and 2) assess the impact of new-onset AF on in-hospital and postdischarge prognosis of patients with sepsis, providing a basis for risk assessment and patient management strategies in sepsis care.

## Methods

### Study population and design

We used the UK Clinical Practice Research Datalink (CPRD) data set, which as of 2018 included 7,998,501 patients in the United Kingdom with linked data of primary care consultation, hospital data (Hospital Episodes Statistics), and death registry data (Office for National Statistics [ONS]).^12^^,^^13^ These data have shown high quality and completeness of clinical information recorded^12^, ^13^, ^14^ and are generally representative of the age, sex, and geographic distribution of the UK population.^15^ Approval for the present study has been obtained upon review by the Medicines and Healthcare products Regulatory Agency Independent Scientific Advisory Committee (17_205). The CPRD data set has ethics approval from the Health Research Authority (21/EM/0265; January 10, 2022).

Our CPRD data set included 5.6 million adult patients age ≥18 years seen from January 1, 1998, to May 30, 2016.^1^ Patients were excluded if they had a prior history of AF before study entry. The time of first diagnosis for sepsis and/or AF was defined as the time of onset, and the time point closest to the start of follow-up was selected. The end date of the study was the earliest of the following: date of death, enrollment cutoff date, last date of primary care data collection, or study end date (May 31, 2016).

Among patients with hospitalization for sepsis and without previous history of AF, we looked at comorbidities that were present prior to, or at the time of, hospitalization. Based on previously published studies,^16^^,^^17^ we considered 12 possible risk factors and incorporated them into our analyses. These were grouped as:

1. Demographic and behavioral characteristics: age at the time of index hospitalization, age >70 years (based on evidence suggesting higher risk of AF in sepsis over 70 years of age^18^), sex, ethnicity (categorized into 2 groups, non-White and White, due to the predominance of White people in the data set), Index of Multiple Deprivation (lower rankings indicating higher levels of socioeconomic deprivation), smoking status (never smokers vs current and former smokers)
2. Common chronic diseases associated with AF: hypertension, diabetes, chronic kidney disease (CKD), valvular disease, ischemic heart disease (IHD), chronic obstructive pulmonary disease (COPD), and heart failure.

The code lists for all diseases, using Reference Exchange Adjudication Documentation and International Classification of Diseases-10 codes from primary and secondary care, were obtained from the Health Data Research UK Phenotype Library,^19^^,^^20^ and are available in Supplemental Table 1.

### Statistical analyses

Baseline characteristics were presented. We reported frequencies (%) for categorical data; and means with SD, or median (IQR) as appropriate for continuous data. Chi-square and *t*-tests were used to examine differences between the compared groups.

Baseline data were compared across patients with sepsis without new-onset AF vs sepsis with new-onset AF utilizing via logistic regression to identify potential risk factors for new-onset AF. Variables with a *P* value of <0.20 in the univariate analysis were subsequently included in the multivariable logistic regression (forward stepwise) to identify which variables were independently associated with new-onset AF in patients with sepsis.

For assessment of in-hospital and long-term outcomes, 2 comparisons were performed: 1) patients with sepsis and new-onset AF vs patients with sepsis without AF; and 2) patients with new-onset AF and sepsis vs patients with new-onset AF without sepsis.

The outcomes of interest were as follows: in-hospital mortality at 7 and 30 days, development of septic shock at 7 and 30 days, length of hospital stay, postdischarge mortality, and stroke. These were assessed separately for the 2 comparisons mentioned above.

Length of hospitalization was calculated as the date of discharge minus the date of admission. Rank sum test was used to analyze mean length of duration. Mortality data were obtained from ONS. In-hospital mortality was defined as mortality occurring during index hospitalization and was assessed separately from postdischarge mortality (ie, mortality in patients surviving the index admission).

Logistic regression was used to assess the OR of 7-day in-hospital mortality and septic shock rates, 30-day in-hospital mortality and septic shock rates. Kaplan-Meier survival curves were traced to illustrate time-to-event for postdischarge mortality and stroke; differences between groups were assessed using the log-rank test. Despite no violation of proportional hazard assumption, landmark analyses were employed for postdischarge stroke in the comparison of patients with new-onset AF with and without sepsis, due to a subtle change in survival curve behavior after the first 3 years of follow-up.

Cox proportional hazards models were used to assess postdischarge mortality and postdischarge stroke. Besides presenting nonadjusted data (Model 1), we adjusted for behavioral and demographic variables, as well as for comorbid conditions (Model 2).

As a sensitivity analysis, we conducted 1:1 propensity score matching analysis using nearest-neighbor matching without replacement. A propensity score was calculated using multivariable logistic regression model including the behavioral and demographic variables, as well as the comorbid conditions in Model 2. In addition, to account for competing risks, we used the Fine-Gray model when mortality was not part of the outcome.

We performed the analyses in the secured Data Safe Haven, meeting the data safety and information governance requirements by University College London, NHS Digital, and ONS. Variable screening and related data processing were performed using R (R Foundation for Statistical Computing) and MySQL software (Oracle Corporation), and statistical analyses were performed using STATA software (StataCorp LLC).

## Results

Our data set comprised 7,691 patients hospitalized for sepsis and new-onset AF, 24,506 patients with sepsis who did not develop new-onset AF, and 95,287 patients hospitalized for new-onset AF who did not have sepsis. Among patients hospitalized with sepsis, 23.9% had new-onset AF. Patients with sepsis without new-onset AF were significantly younger than the 2 other groups. Women and men were equally represented. There was a predominance of White ethnicity, and nearly 3-quarters of patients were smokers or previous smokers and had hypertension. COPD was present in half of patients, and prevalence of CKD, heart failure, or diabetes mellitus ranged between 16 and 37% (Table 1). The number of primary care interactions after discharge was higher in patients with new-onset AF with sepsis compared to those with sepsis without new-onset AF (31.8 ± 10.7 vs 23.3 ± 7.8 interactions per year, *P* < 0.001), as well as in patients with new-onset AF in sepsis compared to those new-onset AF without sepsis (31.8 ± 10.7 vs 24.1 ± 8.0 interactions per year, *P* < 0.001).

**Table 1:** Study Population and Baselines

	Sepsis With New-Onset AF (n = 7,691)	Sepsis Without New-Onset AF (n = 24,506)	New-Onset AF Without Sepsis (n = 95,287)
Age, y	78.25 ± 10.88	69.52 ± 16.85	76.82 ± 11.60
Age >70 y	6,043 (78.57%)	13,403 (54.69%)	71,331 (74.86%)
Female	3,640 (47.33%)	12,780 (52.15%)	47,613 (49.97%)
Non-White ethnicity	181 (2.35%)	1,252 (5.11%)	1,951 (2.05%)
IMD			
1st (most deprived)	1,280 (16.64%)	3,675 (15.00%)	15,559 (16.33%)
2nd	1,577 (20.50%)	5,042 (20.57%)	18,246 (19.15%)
3rd	1,416 (18.41%)	4,644 (18.95%)	18,911 (19.85%)
4th	1,613 (20.97%)	5,310 (21.67%)	20,224 (21.22%)
5th (least deprived)	1,805 (23.47%)	5,835 (23.81%)	22,347 (23.45%)
Region			
East Midlands	167 (2.17%)	612 (2.50%)	2,602 (2.73%)
East of England	841 (10.93%)	2,557 (10.43%)	10,419 (10.93%)
London	941 (12.24%)	3,213 (13.11%)	9,969 (10.46%)
North East	178 (2.31%)	626 (2.55%)	2,036 (2.14%)
North West	1,247 (16.21%)	3,555 (14.51%)	15,426 (16.19%)
South Central	1,039 (13.51%)	3,362 (13.72%)	11,679 (12.26%)
South East Coast	1,243 (16.16%)	3,796 (15.49%)	13,425 (14.09%)
South West	917 (11.92%)	2,825 (11.53%)	13,995 (14.69%)
West Midlands	838 (10.90%)	2,863 (11.68%)	12,029 (12.62%)
Yorkshire & The Humber	280 (3.64%)	1,097 (4.48%)	3,707 (3.89%)
Smokers	6,351 (82.58%)	18,159 (74.10%)	76,250 (80.02%)
CKD	2,199 (28.59%)	4,283 (17.48%)	15,265 (16.02%)
COPD	4,337 (56.39%)	11,286 (46.05%)	44,357 (46.55%)
Diabetes mellitus	2,181 (28.36%)	5,729 (23.38%)	15,944 (16.73%)
Heart failure	2,881 (37.46%)	3,981 (16.25%)	21,905 (22.99%)
Hypertension	5,833 (75.84%)	13,982 (57.06%)	63,414 (66.55%)
Ischemic heart disease	152 (1.98%)	192 (0.78%)	1,034 (1.09%)
Valvular heart disease	766 (9.96%)	977 (3.99%)	7,422 (7.79%)
Charlson comorbidity index	4.64 (3.05)	4.15 (3.18)	2.69 (2.47)
Oral anticoagulant or antiplatelet use after discharge	2,733 (35.54%)	–	53,910 (56.58%)

Values are mean ± SD or n (%).

AF = atrial fibrillation; CKD = chronic kidney disease; COPD = chronic obstructive pulmonary disease; IMD = Index of Multiple Deprivation.

In univariate logistic regression, all 12 variables were associated with increased odds of new-onset AF in setting of sepsis. All 12 variables were included in the multivariate logistic regression model. All variables except diabetes mellitus were significantly associated with the development of new-onset AF in patients hospitalized with sepsis: age 70 years, White ethnicity, smokers, CKD, COPD, heart failure, hypertension, IHD, and valvular heart disease were associated with increased risk, and female sex and being from a less socioeconomic deprived quintile were protective (Table 2).

**Table 2:** Independent Risk Factors for Incident AF in Sepsis

	Univariate		Multivariable	
	Odds Ratio (95% CI)	*P* Value	Odds Ratio (95% CI)	*P* Value
Age >70 y	3.04 (2.86-3.23)	<0.001	2.17 (2.04-2.31)	<0.001
Female	0.82 (0.78-0.87)	<0.001	0.85 (0.80-0.90)	<0.001
White ethnicity	2.23 (1.91-2.62)	<0.001	1.72 (1.46-2.03)	<0.001
IMD			-	-
1st (most deprived)	Reference	-	Reference	-
2nd	0.90 (0.82-0.98)	0.013	0.91 (0.83-1.00)	0.044
3rd	0.88 (0.80-0.96)	0.003	0.89 (0.81-0.98)	0.012
4th	0.87 (0.80-0.95)	0.002	0.85 (0.78-0.93)	<0.001
5th (least deprived)	0.89 (0.82-0.96)	0.005	0.88 (0.80-0.96)	0.003
Smokers	1.66 (1.55-1.77)	<0.001	1.38 (1.29-1.48)	<0.001
CKD	1.89 (1.78-2.01)	<0.001	1.20 (1.13-1.28)	<0.001
COPD	1.51 (1.44-1.59)	<0.001	1.17 (1.10-1.23)	<0.001
Diabetes mellitus	1.30 (1.22-1.37)	<0.001	0.99 (0.93-1.06)	0.858
Heart failure	3.09 (2.92-3.27)	<0.001	2.14 (2.01-2.27)	<0.001
Hypertension	2.36 (2.23-2.50)	<0.001	1.54 (1.45-1.65)	<0.001
Ischemic heart disease	2.55 (2.06-3.16)	<0.001	1.62 (1.29-2.03)	<0.001
Valvular heart disease	2.66 (2.41-2.94)	<0.001	1.69 (1.53-1.88)	<0.001

Abbreviations as in Table 1.

### Duration of hospital stay and in-hospital mortality

The mean duration of hospital stay in patients with new-onset AF in the context of sepsis was 24.70 ± 0.32 days, which was longer than for patients with sepsis who did not develop AF (20.44 ± 0.19 days) and patients hospitalized for new-onset AF without sepsis (20.89 ± 0.09 days). Differences were significant for both comparisons (*P* < 0.001).

Unadjusted and adjusted analyses for the following outcomes were reported in Tables 3 and 4. In-hospital mortality at 7 days was observed for 1,153 (14.99%) patients with sepsis and new-onset AF, 1,261 (5.15%) patients with sepsis without new-onset AF, and for 4,466 (4.69%) patients hospitalized with new-onset AF without sepsis. Patients with sepsis and new-onset AF had a greater risk of in-hospital 7-day mortality compared to those with sepsis who did not develop new-onset AF (adjusted OR: 2.25; 95% CI: 2.06-2.46).

**Table 3:** Mortality and Cardiovascular Outcomes Postdischarge

	Comparison A New-Onset AF in Sepsis vs Sepsis Without New-Onset AF		Comparison B New-Onset AF in Sepsis vs New-Onset AF Without Sepsis	
	OR (95% CI)	*P* Value	OR (95% CI)	*P* Value
In-hospital mortality in 7 d				
Unadjusted	3.25 (2.99-3.54)	<0.001	1.58 (1.44-1.73)	<0.001
Adjusted^a^	2.25 (2.06-2.46)	<0.001	1.46 (1.32-1.60)	<0.001
In-hospital mortality in 30 d				
Unadjusted	4.10 (3.84-4.38)	<0.001	1.54 (1.45-1.64)	<0.001
Adjusted^a^	2.91 (2.72-3.12)	<0.001	1.44 (1.36-1.54)	<0.001

	**HR (95% CI)**		**HR (95% CI)**	

Mortality postdischarge				
Cox unadjusted	1.44 (1.38-1.49)	<0.001	1.61 (1.57-1.65)	<0.001
Cox adjusted^a^	1.07 (1.03-1.12)	0.001	1.45 (1.41-1.49)	<0.001
Stroke postdischarge				
Cox unadjusted	1.82 (3.40-3.94)	<0.001	1.23 (1.14-1.32)	<0.001
Cox adjusted^a^	1.19 (1.08-1.32)	0.001	1.16 (1.08-1.24)	<0.001
Fine-Gray unadjusted	1.61 (1.48-1.76)	<0.001	1.12 (1.05-1.20)	0.001
Fine-Gray adjusted^a^	1.18 (1.08-1.30)	<0.001	1.07 (1.00-1.14)	0.062
Heart failure hospitalization postdischarge				
Cox unadjusted	3.65 (3.39-3.92)	<0.001	1.68 (1.60-1.76)	<0.001
Cox adjusted^a^	1.79 (1.66-1.93)	<0.001	1.43 (1.36-1.50)	<0.001
Fine-Gray unadjusted	3.20 (2.98-3.44)	<0.001	1.64 (1.56-1.72)	<0.001
Fine-Gray adjusted^a^	1.81 (1.67-1.97)	<0.001	1.45 (1.37-1.52)	<0.001
MI hospitalization postdischarge				
Cox unadjusted	2.31 (2.10-2.53)	<0.001	1.50 (1.40-1.61)	<0.001
Cox adjusted^a^	1.23 (1.12-1.36)	<0.001	1.26 (1.17-1.35)	<0.001
Fine-Gray unadjusted	1.95 (1.78-2.13)	<0.001	1.46 (1.36-1.56)	<0.001
Fine-Gray adjusted^a^	1.25 (1.13-1.38)	<0.001	1.17 (1.09-1.26)	<0.001

MI = myocardial infarction.

a Adjusted for age, sex, race, IMD, smoke status, and comorbidities: diabetes mellitus, hypertension, heart failure, COPD, CKD, valvular disease, IHD, and Charlson comorbidity index.

**Table 4:** In-Hospital Septic Shock in New-Onset AF With Sepsis vs Sepsis Without New-Onset AF

	OR (95% CI)	*P* Value
In-hospital septic shock in 7 d		
Unadjusted	1.56 (1.20-2.01)	0.001
Adjusted^a^	1.38 (1.05-1.81)	0.020
In-hospital septic shock in 30 d		
Unadjusted	1.54 (1.19-1.98)	<0.001
Adjusted^a^	1.37 (1.04-1.79)	0.022

Abbreviation as in Table 1.

a Adjusted for age, sex, race, IMD, smoke status and comorbidities: diabetes mellitus, hypertension, heart failure, COPD, CKD, valvular disease, IHD, and Charlson comorbidity index.

When comparing patients with sepsis and new-onset AF vs patients hospitalized with new-onset AF but without sepsis, 7-day in-hospital mortality was higher in the AF with sepsis (adjusted OR: 1.46; 95% CI: 1.32-1.60) (Table 3).

In-hospital mortality at 30 days occurred in 2,292 (29.80%) of patients with sepsis and new-onset AF, 2,299 (9.38%) patients with sepsis who did not develop AF, and in 12,063 (12.66%) patients hospitalized with new-onset AF who did not have sepsis. Mortality was higher in patients with both new-onset AF and sepsis when compared to those with sepsis only (adjusted OR: 2.91; 95% CI: 2.72, 3.12), and when compared to those hospitalized with new-onset AF and no sepsis (adjusted OR: 1.44; 95% CI: 1.36, 1.54) (Table 3).

When compared to patients with sepsis without AF, patients with new-onset AF during sepsis had increased risk of septic shock at 7 days (1.14% vs 0.74%; adjusted OR: 1.38; 95% CI: 1.05-1.81) and 30 days (1.16% vs 0.75%; adjusted OR: 1.37; 95% CI: 1.04-1.79) (Table 4).

### Survival postdischarge

**Figure 1:**
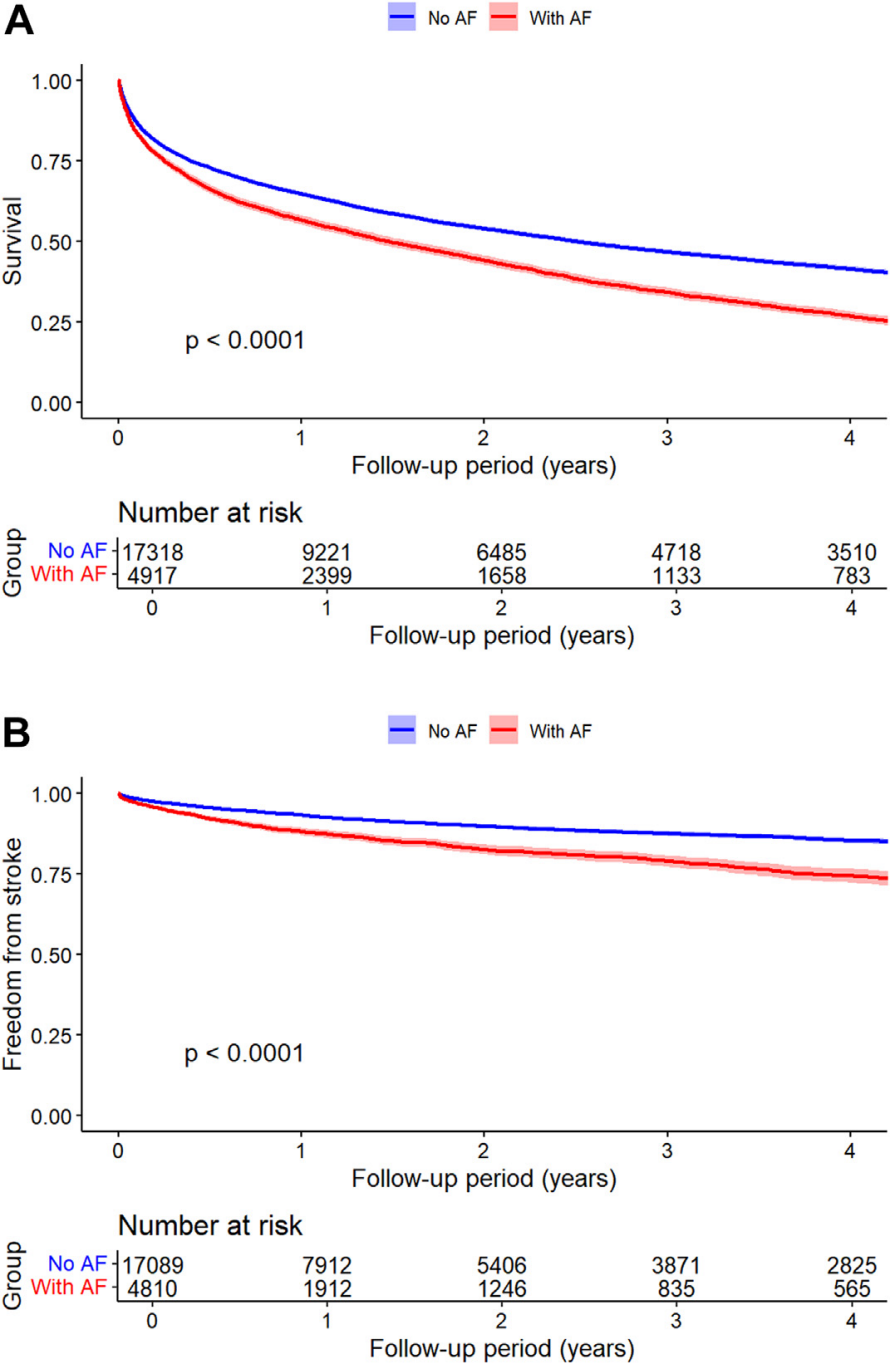
Postdischarge Outcomes for the Comparison of Patients With Sepsis and New-Onset AF vs Sepsis Without AF (A) Mortality. (B) Stroke. AF = atrial fibrillation.

**Figure 2:**
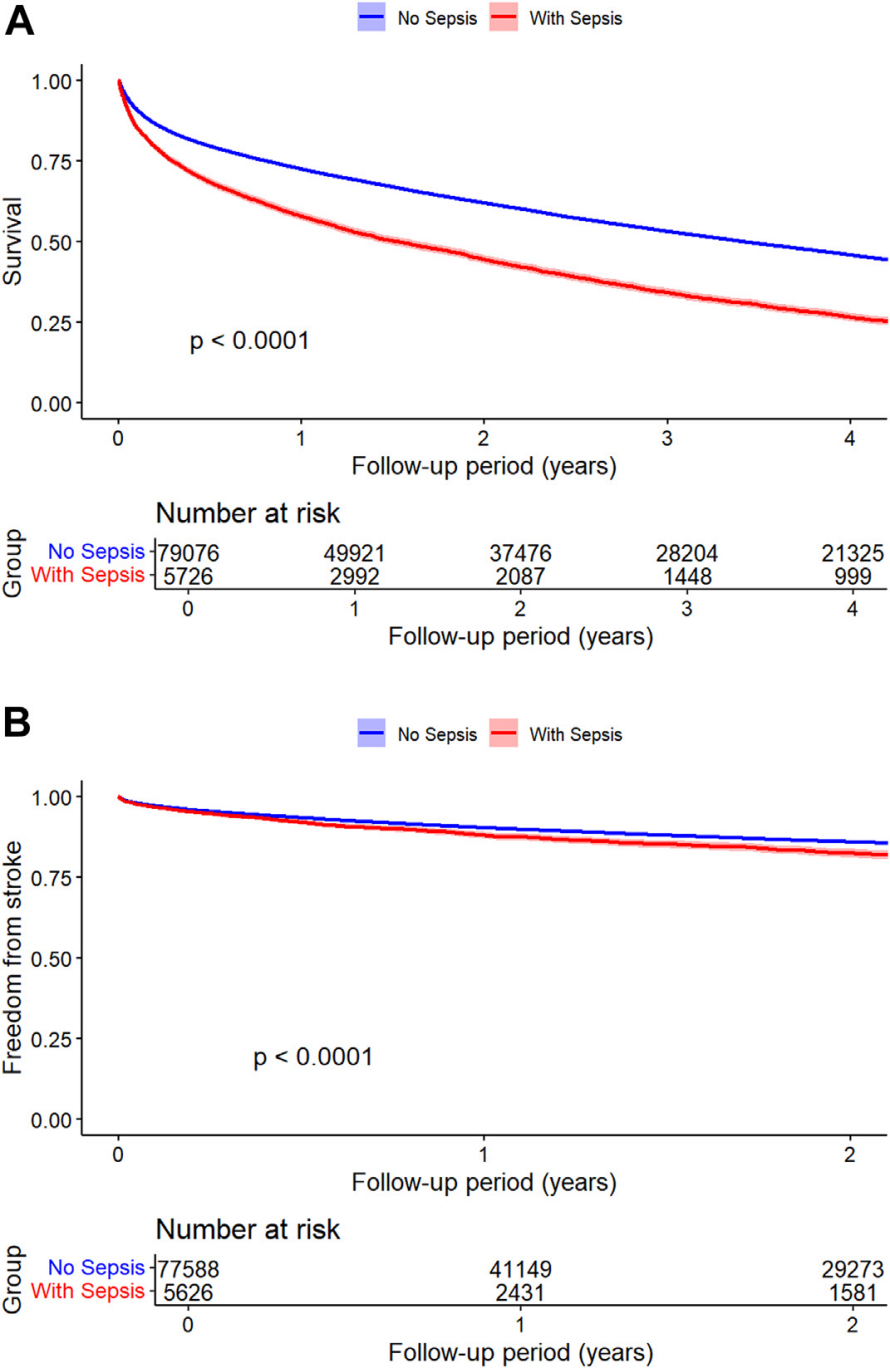
Postdischarge Outcomes for the Comparison of Patients With Sepsis and New-Onset AF vs Patients Hospitalized With New-Onset AF Without Sepsis (A) Mortality. (B) Stroke. Abbreviation as in Figure 1.

Median survival time postdischarge was 1.48 years for patients with sepsis and new-onset AF, 2.49 years for patients with sepsis who did not develop AF, and 3.41 years for patients hospitalized with new-onset AF who did not have sepsis (Figures 1 and 2 show the survival curves for this endpoint). Patients with sepsis and new-onset AF who survived to hospital discharge had higher mortality during follow-up than those who survived sepsis and did not develop AF during the admission (72.02% vs 44.93%; adjusted HR: 1.07; 95% CI: 1.03-1.12; *P* = 0.001). Compared with patients hospitalized with new-onset AF who did not have sepsis, patients with both new-onset AF and sepsis had a higher risk mortality risk during follow-up (72.02% vs 58.59%; adjusted HR: 1.45; 95% CI: 1.41-1.49).

### Postdischarge stroke, heart failure, and myocardial infarction

Postdischarge stroke occurred more frequently in patients with sepsis and new-onset AF when compared with patients with sepsis who did not have AF (13.43% vs 7.15%; adjusted HR: 1.18; 95% CI: 1.08-1.30) (Figure 1). For the comparison of patients with sepsis and new-onset AF vs patients hospitalized with new-onset AF without sepsis, landmark analysis was utilized for calculation of the HR and showed a trend for more frequent postdischarge stroke in the group with sepsis (adjusted HR: 1.07; 95% CI: 1.00-1.14). Survival curves for incidence of stroke since discharge show that the 2 groups diverge in the initial follow-up period (Figure 2).

Postdischarge hospitalizations for heart failure and myocardial infarction were more frequent in patients with sepsis and new-onset AF when compared with sepsis and who did not have AF (adjusted HR: 1.81; 95% CI: 1.67-1.97 for heart failure, and 1.25, 95% CI: 1.13-1.38 for myocardial infarction) and new-onset AF without sepsis (adjusted HR: 1.45; 95% CI: 1.37-1.52 for heart failure, and 1.17, 95% CI: 1.09-1.26 for myocardial infarction) (Table 3, Supplemental Figures 1 and 2).

### Sensitivity analysis

Supplemental Table 2 shows the baseline characteristics after propensity score matching. Using propensity score matching, findings similar to those in the primary analyses were observed (Supplemental Tables 3 and 4), except for postdischarge stroke when comparing new-onset AF with sepsis to new-onset AF without sepsis where no significant differences were observed (adjusted HR: 1.04; 95% CI: 0.95-1.13; *P* = 0.445).

## Discussion

Utilizing a nationwide UK data set, we described the association of sepsis with new-onset AF, covering both the initial hospitalization and the postdischarge period. We observed that one-quarter of patients hospitalized with sepsis developed new-onset AF and identified risk factors independently associated with the development of arrhythmia. Advanced age, male sex, White ethnicity, socioeconomic deprivation, smoking status, presence of COPD, heart failure, IHD, valvular heart disease, and hypertension were all associated with new-onset AF. Patients with sepsis who developed AF had worse in-hospital prognosis (ie, increased duration of hospitalization, risk of septic shock, and mortality), and were more likely to die postdischarge when compared to patients hospitalized with sepsis who did not develop AF, or to patients hospitalized with AF who did not have sepsis. Finally, patients with sepsis and new-onset AF experienced higher rates of stroke, heart failure, and myocardial infarction postdischarge than patients with sepsis who did not develop the arrhythmia. Our analysis, ranging for a period of nearly 2 decades, suggests underutilization of oral anticoagulants, more pronounced in the group of patients with new-onset AF in the setting of sepsis (Central Illustration).

**Central Illustration:**
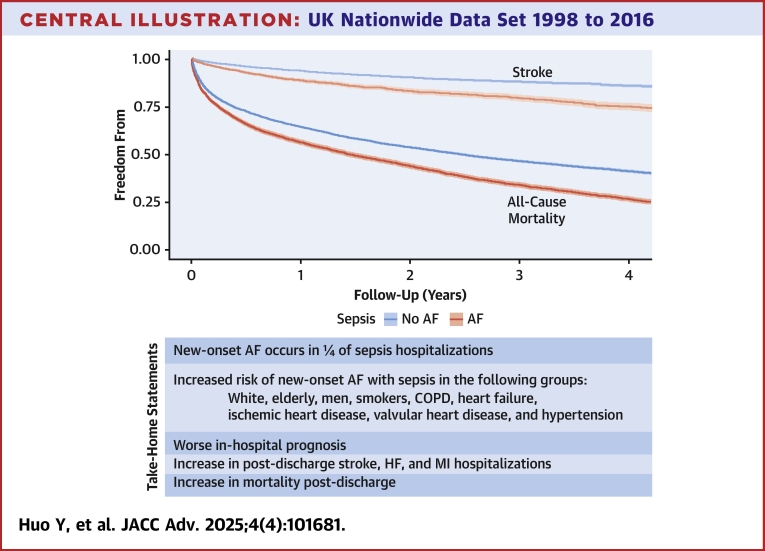
UK Nationwide Data Set 1998 to 2016 COPD = chronic obstructive pulmonary disease; HF = heart failure; MI = myocardial infarction; other abbreviation as in Figure 1.

The rate of new-onset AF in our study is 4 times higher than what has been described using U.S. claims data,^7^^,^^21^ but is comparable to what has been reported in one multicenter Dutch study.^11^ The detected high rate of AF reinforces the need for actively monitoring these patients, and establishing a diagnosis as, as shown in our analyses, the AF diagnosis has prognostic implications beyond the index admission. Furthermore, a better understanding of who are the patients at risk may help designing future trials assessing strategies to prevent the development of arrhythmia and potentially improve patient-related outcomes.

Our data demonstrate for the first time that comorbidities like COPD, and hypertension, IHD, and valvular heart disease are also associated with increased risk of new-onset AF in the sepsis population. This goes beyond previously described factors like advanced age, female sex, and White ethnicity,^7^^,^^21^ and aligns with current knowledge for AF risk factors in the general population. Hypertension, history of myocardial infarction, and heart failure are part of the CHARGE-AF (Cohorts for Heart and Aging Research in Genomic Epidemiology-Atrial Fibrillation) risk score^22^ and multiple other risk schemes^23^ predicting AF development. Supporting evidence for the association between COPD and development of AF in the general population is also robust, and includes a systematic review of 12 observational studies,^24^ and 2 risk prediction schemes the MHS (Morbidity, Hospitalization, and Stroke)^25^ and C_2_HEST (Congestive heart failure, Hypertension, Elderly (age >75 years), Stroke/TIA, and vascular disease)^26^ scores). Interestingly, a very low rate of new-onset AF has been described among patients hospitalized with sepsis in Taiwan^27^ and China,^28^ which agrees with our findings on ethnicity as an important driver for new-onset AF in sepsis.

The length of hospitalization in AF patients with sepsis was significantly longer than for patients without the 2 conditions. In our study, the mean number in days in hospital was similar to the one described by Guenancia and colleagues,^29^ but shorter than reported by Christian et al.^30^ Furthermore, we observed a 40% higher rate of septic shock in patients with new-onset AF and sepsis, supporting previous reports of higher severity of sepsis in the AF population from previous studies,^31^ which may explain why these patients have higher mortality during hospitalization.^21^

Similarly to Walkey et al,^7^ we observed a high mortality during follow-up (approximately 75% at 5 years) in patients with sepsis and new-onset AF, which was higher than for patients with sepsis without AF. On the other hand, we observed a much higher rate of stroke at 5 years (nearly 20% based on survival curves) than the aforementioned study,^7^ and more in line with the rate observed by Ayaz et al.^32^

Patients with sepsis may be at increased risk of myocardial stress and AF due to the release of pro-inflammatory cytokines, changes in vascular volume, high stress hormones, autonomic dysfunction, and cardiovascular injury. These inflammatory responses may cause myocardial electrophysiological instability and necrosis, which in turn may induce various arrhythmias, especially AF.^33^ The systemic inflammatory response may also trigger a coagulopathic state, leading to stroke.^34^ However, our findings suggest that AF in the sepsis population represents more than a stress response during infection and signals the presence of an important prothrombotic risk state that remains even after treatment of the index insult. Of relevance, use of anticoagulants in our AF cohort was low (<1/3 as per our previous publication^1^) as most patients were hospitalized prior to the publication of the CHA_2_DS_2_VASc score and the availability of direct oral anticoagulants in the United Kingdom.

Our findings reinforce the need to actively screen AF and risk stratify AF patients with sepsis so they can receive appropriate treatment. Adding to this matter, a Danish nationwide study has investigated the broader problem of infection-related new-onset AF, similarly to sepsis, and increased thromboembolic risk was observed for infection-related AF, prompting the need for risk stratification and potential use of anticoagulation in AF arising in the setting of any infectious process.^35^ Our observations are of interest for local agencies like the UK's National Institute for Health and Care Excellence but could also provide support to other international AF and sepsis guidelines.

Our study has some limitations that need to be highlighted. First, these are UK data, representing a majority of Caucasian elderly patients, and may not apply to countries with a different demographic structure. Second, as for every electronic health records investigation, it is possible some AF cases may have been misclassified due to coding. However, we have utilized a validated AF definition,^19^^,^^20^ which has previously reproduced results from Framingham Heart Study and other landmark studies with clinical adjudication.^1^ Finally, as for every observational analysis, we cannot infer causality and there is the risk of unmeasured risk factors or comorbidities. We overcame this limitation by including key comorbidities supported by the previous literature.^16^^,^^17^^,^^31^

## Conclusions

AF during sepsis is common and is not an innocent finding. Some population groups are at higher risk of developing this arrhythmia, such as White elderly individuals with smoking history and/or history or COPD, hypertension, and diabetes mellitus.

Active monitoring should be pursued as AF has important short- and long-term prognostic implications and requires appropriate management for preventing thromboembolic complications and mortality.

## Funding Support and Author Disclosures

Dr Providencia is supported by the UCL BHF Research Accelerator AA/18/6/34223, 10.13039/501100000272NIHR grant NIHR129463, and UKRI/ERC/HORIZON 10103153 Aristoteles. Dr Lip is a consultant and speaker for BMS/Pfizer, Boehringer Ingelheim, Daiichi Sankyo, and Anthos; is a National Institute for Health and Care Research (NIHR) senior investigator and co-PI of the AFFIRMO project on multimorbidity in AF (grant agreement No 899871), TARGET project on digital twins for personalized management of atrial fibrillation and stroke (grant agreement No 101136244) and ARISTOTELES project on artificial intelligence for management of chronic long-term conditions (grant agreement No 101080189), which are all funded by the EU's Horizon Europe Research & Innovation programme. All other authors have reported that they have no relationships relevant to the contents of this paper to disclose.

## References

[1] Chung S.C., Sofat R., Acosta-Mena D. (2021). Atrial fibrillation epidemiology, disparity and healthcare contacts: a population-wide study of 5.6 million individuals. Lancet Reg Health Eur.

[2] Chung S.C., Schmit A.F., Lip G.Y.H., Providencia R. (2023). Electronic health record-wide association study for atrial fibrillation in a British cohort. Front Cardiovasc Med.

[3] Hindricks G., Potpara T., Dagres N., ESC Scientific Document Group (2021). 2020 ESC Guidelines for the diagnosis and management of atrial fibrillation developed in collaboration with the European Association for Cardio-Thoracic Surgery (EACTS): the Task Force for the diagnosis and management of atrial fibrillation of the European Society of Cardiology (ESC) Developed with the special contribution of the European Heart Rhythm Association (EHRA) of the ESC. Eur Heart J.

[4] Singer M., Deutschman C.S., Seymour C.W. (2016). The third international consensus definitions for sepsis and septic shock (Sepsis-3). JAMA.

[5] Rudd K.E., Johnson S.C., Agesa K.M. (2020). Global, regional, and national sepsis incidence and mortality, 1990-2017: analysis for the Global Burden of Disease Study. Lancet.

[6] An Y., Ogawa H., Yamashita Y. (2019). Causes of death in Japanese patients with atrial fibrillation: the fushimi atrial fibrillation registry. Eur Heart J Qual Care Clin Outcomes.

[7] Walkey A.J., Hammill B.G., Curtis L.H., Benjamin E.J. (2014). Long-term outcomes following development of new-onset atrial fibrillation during sepsis. Chest.

[8] Salman S., Bajwa A., Gajic O., Afessa B. (2008). Paroxysmal atrial fibrillation in critically ill patients with sepsis. J Intensive Care Med.

[9] Kanji S., Williamson D.R., Yaghchi B.M., Albert M., McIntyre L., Canadian Critical Care Trials Group (2012). Epidemiology and management of atrial fibrillation in medical and noncardiac surgical adult intensive care unit patients. J Crit Care.

[10] Moss T.J., Calland J.F., Enfield K.B. (2017). New-onset atrial fibrillation in the critically ill. Crit Care Med.

[11] Klein Klouwenberg P.M.C., Frencken J.F., Kuipers S., MARS consortium (2017). Incidence, predictors, and outcomes of new-onset atrial fibrillation in critically ill patients with sepsis. A cohort study. Am J Respir Crit Care Med.

[12] Walley T., Mantgani A. (1997). The UK general practice research database. Lancet.

[13] Gallagher A.M., Dedman D., Padmanabhan S., Leufkens H.G.M., de Vries F. (2019). The accuracy of date of death recording in the clinical practice research datalink GOLD database in England compared with the office for national statistics death registrations. Pharmacoepidemiol Drug Saf.

[14] Padmanabhan S., Carty L., Cameron E., Ghosh R.E., Williams R., Strongman H. (2019). Approach to record linkage of primary care data from clinical practice research datalink to other health-related patient data: overview and implications. Eur J Epidemiol.

[15] Herrett E., Thomas S.L., Schoonen W.M., Smeeth L., Hall A.J. (2010). Validation and validity of diagnoses in the general practice research database: a systematic review. Br J Clin Pharmacol.

[16] Li J., Agarwal S.K., Alonso A. (2014). Airflow obstruction, lung function, and incidence of atrial fibrillation: the Atherosclerosis Risk in Communities (ARIC) study. Circulation.

[17] Odutayo A., Wong C.X., Hsiao A.J., Hopewell S., Altman D.G., Emdin C.A. (2016). Atrial fibrillation and risks of cardiovascular disease, renal disease, and death: systematic review and meta-analysis. BMJ.

[18] Fathi M., Markazi-Moghaddam N., Ramezankhani A. (2019). A systematic review on risk factors associated with sepsis in patients admitted to intensive care units. Aust Crit Care.

[19] Denaxas S., Gonzalez-Izquierdo A., Direk K. (2019). UK phenomics platform for developing and validating electronic health record phenotypes: CALIBER. J Am Med Inform Assoc.

[20] Health Data Research UK (2024). HDRUK Phenotype library. https://phenotypes.healthdatagateway.org/.

[21] Walkey A.J., Wiener R.S., Ghobrial J.M., Curtis L.H., Benjamin E.J. (2011). Incident stroke and mortality associated with new-onset atrial fibrillation in patients hospitalized with severe sepsis. JAMA.

[22] Alonso A., Krijthe B.P., Aspelund T. (2013). Simple risk model predicts incidence of atrial fibrillation in a racially and geographically diverse population: the CHARGE-AF consortium. J Am Heart Assoc.

[23] Goudis C., Daios S., Dimitriadis F., Liu T. (2023). CHARGE-AF: a useful score for atrial fibrillation prediction?. Curr Cardiol Rev.

[24] Xue Z., Guo S., Liu X. (2022). Impact of COPD or asthma on the risk of atrial fibrillation: a systematic review and meta-analysis. Front Cardiovasc Med.

[25] Aronson D., Shalev V., Katz R., Chodick G., Mutlak D. (2018). Risk score for prediction of 10-year atrial fibrillation: a community-based study. Thromb Haemost.

[26] Li Y.G., Pastori D., Farcomeni A. (2019). A simple clinical risk score (C2HEST) for predicting incident atrial fibrillation in asian subjects. Chest.

[27] Cheng C.A., Cheng C.G., Lin H.C. (2017). New-onset atrial fibrillation-related ischemic stroke occurring after hospital discharge in septicemia survivors. QJM.

[28] Liu Y.W., Wang Y.F., Chen Y. (2024). A nationwide study on new onset atrial fibrillation risk factors and its association with hospital mortality in sepsis patients. Sci Rep.

[29] Guenancia C., Binquet C., Laurent G. (2015). Incidence and predictors of new-onset atrial fibrillation in septic shock patients in a medical ICU: data from 7-day Holter ECG monitoring. PLoS One.

[30] Christian S.A., Schorr C., Ferchau L., Jarbrink M.E., Parrillo J.E., Gerber D.R. (2008). Clinical characteristics and outcomes of septic patients with new-onset atrial fibrillation. J Crit Care.

[31] Xiao F.P., Chen M.Y., Wang L. (2021). Outcomes of new-onset atrial fibrillation in patients with sepsis: a systematic review and meta-analysis of 225,841 patients. Am J Emerg Med.

[32] Ayaz A., Ibrahim M., Arshad A. (2024). Long-term risk of stroke after new-onset atrial fibrillation in sepsis survivors: a 2-year follow-up study. J Intensive Care Med.

[33] Steinberg I., Brogi E., Pratali L. (2019). Atrial fibrillation in patients with septic shock: a one-year observational pilot study. Turk J Anaesthesiol Reanim.

[34] Annane D., Sébille V., Duboc D. (2008). Incidence and prognosis of sustained arrhythmias in critically ill patients. Am J Respir Crit Care Med.

[35] Gundlund A., Olesen J.B., Butt J.H. (2020). One-year outcomes in atrial fibrillation presenting during infections: a nationwide registry-based study. Eur Heart J.

