# Supplementary material for: Risk Factors and Prognosis of New-Onset Atrial Fibrillation in Sepsis: A Nationwide Electronic Health Record Study
Source: JACC Adv. 2025 Apr 23;4(4):101681. doi: 10.1016/j.jacadv.2025.101681 (PMC12139237; doi:10.1016/j.jacadv.2025.101681)
Supplement: Supplementary data [file mmc1.docx]

**Supplemental Figure 1.** Heart failure and myocardial infarction hospitalizations post-discharge for the comparison of patients with sepsis and new-onset AF vs. sepsis without AF.


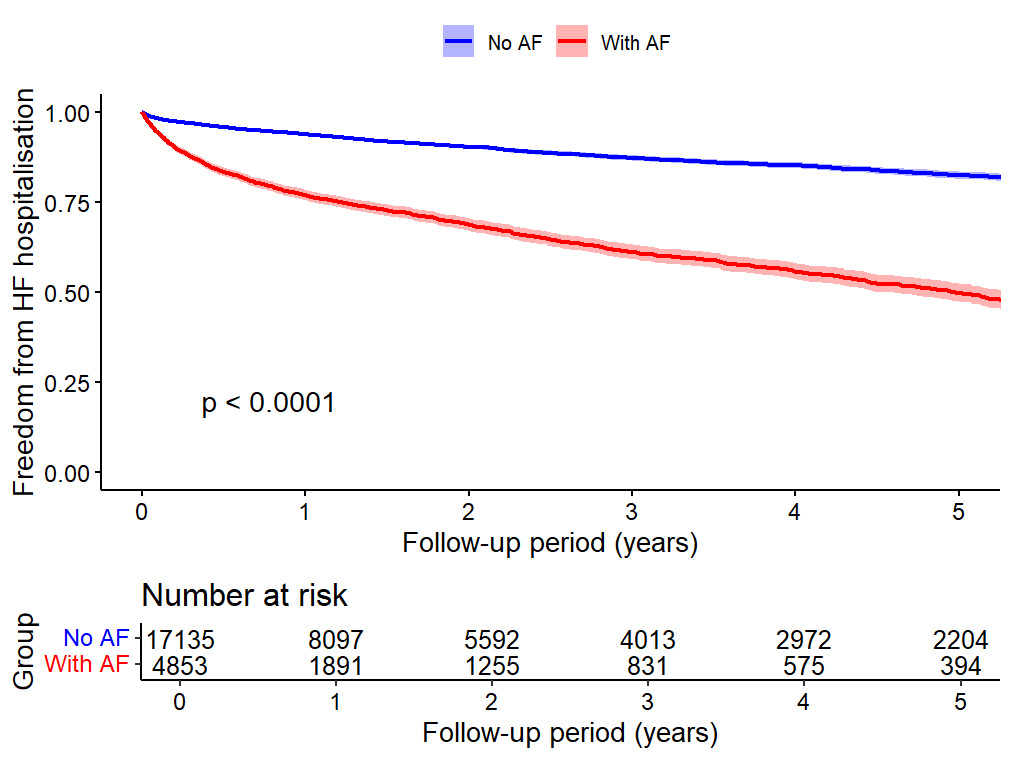


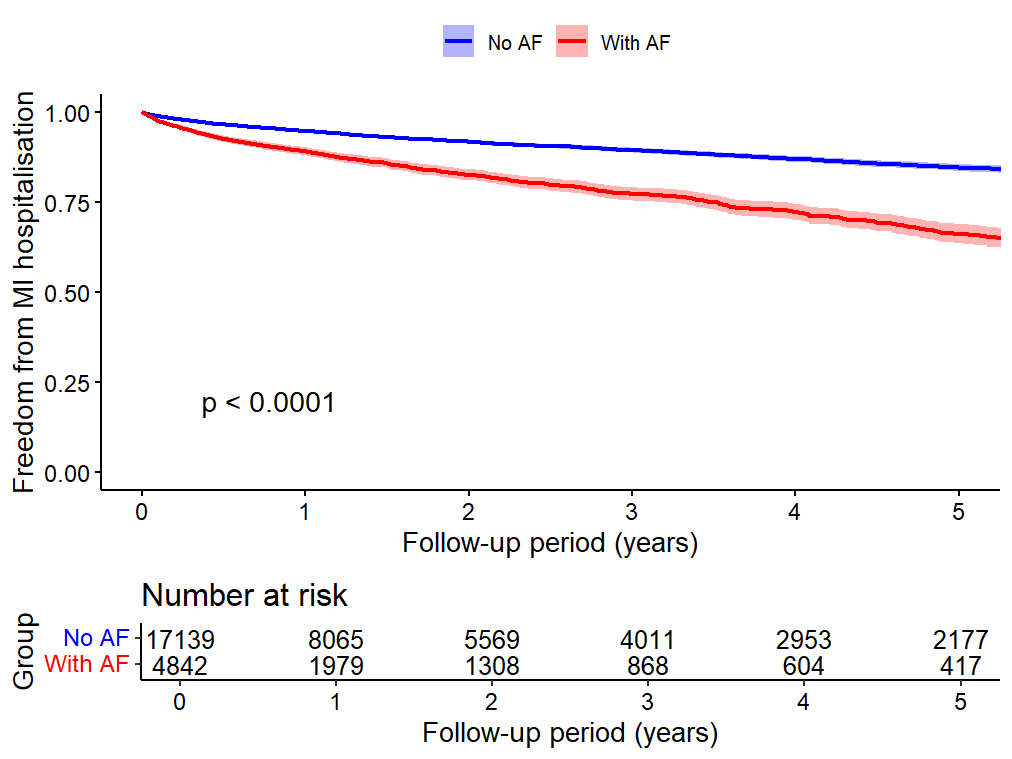


**Supplemental Figure 2.** Heart failure and myocardial infarction hospitalizations post-discharge for the comparison of patients with sepsis and new-onset AF vs. patients hospitalized with new-onset AF without sepsis


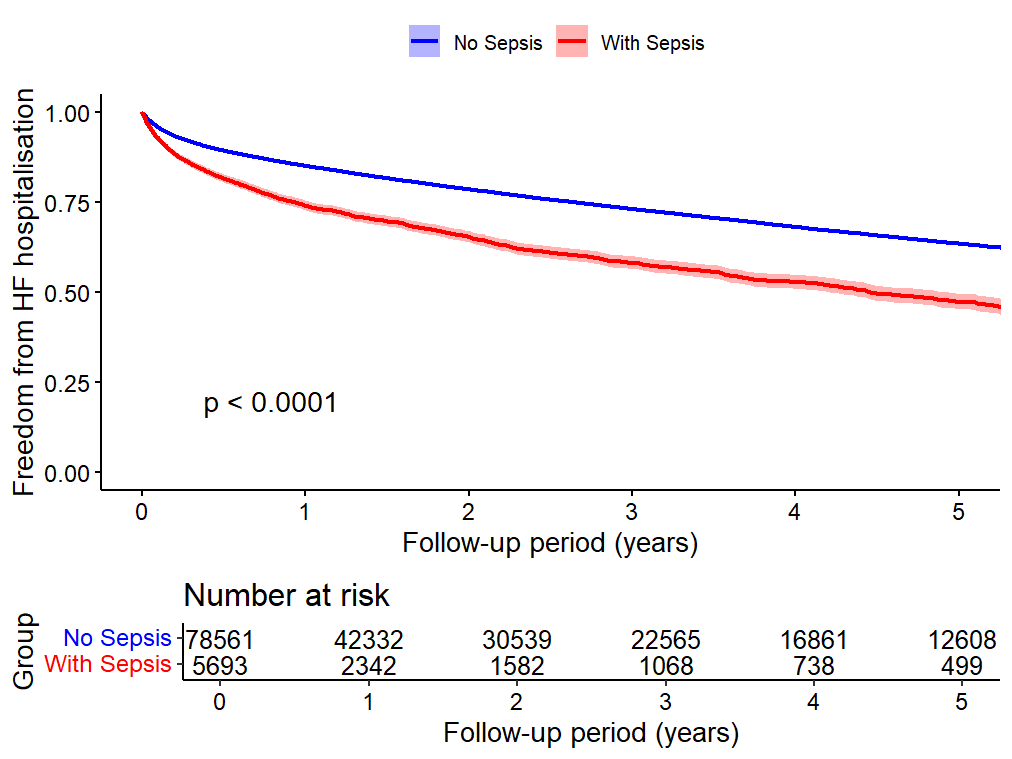


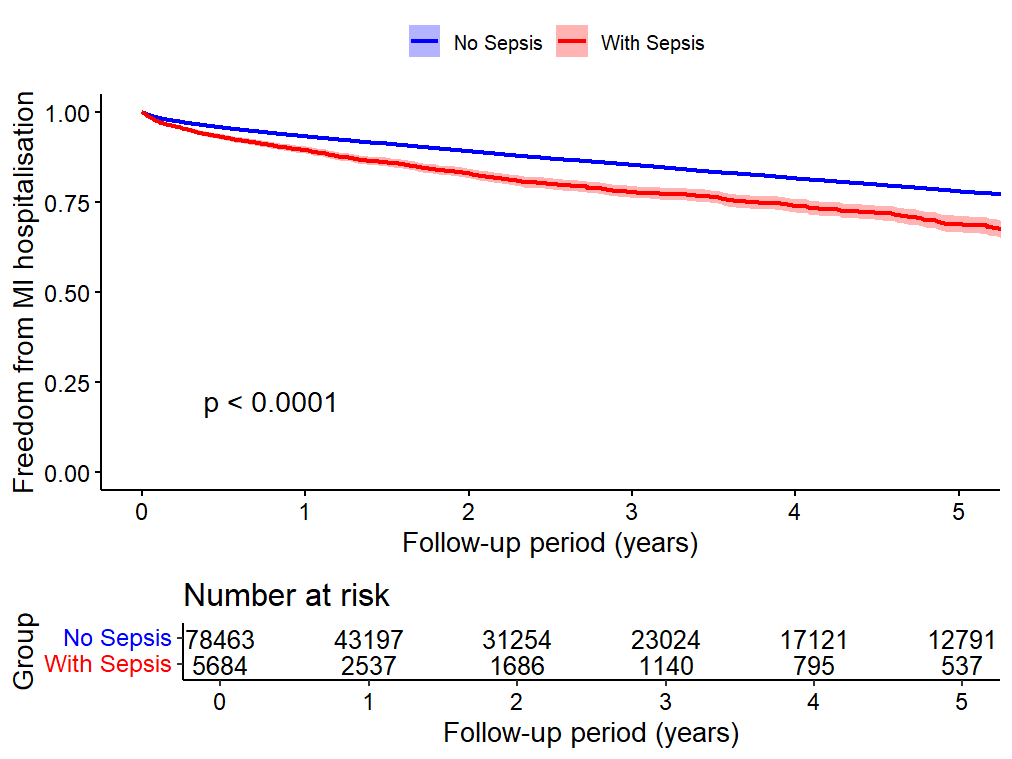


**Supplemental Table 1.** Clinical codes used for defining comorbidities in the study

| **Exposure and covariates** | **Definitions** |
| --- | --- |
| AF | ICD10: I48  Read codes: G573400, G573500, 3272.00, G573000, G573300, G573.00, G573z00 |
| Sepsis | ICD10: A39, A40, A41  Read codes: A02.., A40.., A41.., 1JX.. |
| Smoking status | ICD10: F17, Z716, and Z720  Read codes: 1371, 1371.11, 137L.00, 13WK.00, 9kn..00, 1377, 1378, 1379, 137A.00, 137B.00, 137F.00, 137K.00, 137N.00, 137O.00, 137S.00, 137T.00, 137j.00, 9km..00, E251300, H310100, ZV11600 |
| Diabetes | ICD10: E13, E14, G590, G632, H280, H360, M142, N083  Read codes: C108.12, C108.13, C108011, C108012, C108211, C108212, C108411, C108412, C108511, C108512, C108711, C108712, C108811, C108812, C108911, C108912, C108A11, C108D11, C108E11, C108E12, C108F11, C108H11, C108J11, C108J12, C10E.00, C10E.11, C10E000, C10E100, C10E200, C10E300, C10E311, C10E400, C10E411, C10E500, C10E511, C10E600, C10E700, C10E711, C10E800, C10E900, C10E911, C10EA00, C10EA11, C10EB00, C10EC00, C10EC11, C10ED00, C10EE00, C10EF00, C10EG00, C10EH00, C10EJ00, C10EK00, C10EL00, C10EM00, C10EM11, C10EN00, C10EN11, C10EP00, C10EP11, C10EQ00, C109.12, C109.13, C109011, C109012, C109111, C109112, C109211, C109212, C109411, C109412, C109511, C109512, C109611, C109612, C109711, C109712, C109A11, C109B11, C109C11, C109C12,C109D11, C109D12, C109E11, C109E12, C109F11, C109F12, C109G11, C109G12, C109H11, C109H12, C109J00, C109J12, C109K00, C10F.00, C10F.11, C10F000, C10F011, C10F100, C10F200, C10F211, C10F300, C10F311, C10F400, C10F411, C10F500, C10F600, C10F611, C10F700, C10F711, C10F900, C10F911, C10FA00, C10FA11, C10FB00, C10FB11, C10FC00, C10FD00, C10FD11, C10FE00, C10FE11, C10FF00, C10FG00, C10FH00, C10FJ00, C10FJ11, C10FK00, C10FL00, C10FL11, C10FM00, C10FM11, C10FN00, C10FP00. C10FQ00, C10FR00 |
| Hypertension | ICD10: I10, I11, I12, I13, I14, I15  Read codes: 14A2.00, 2126100, 212K.00, 9OI9.00, 6624, 6627, 6628, 662F.00, 662G.00, 662O.00, 662b.00, 662c.00, 662d.00  662r.00, 7Q01.00, 8B26.00, 8BL0.00, 8I3N.00, F404200, F421300, G2...00, G2...11, G20..00, G200.00, G201.00, G202.00, G203.00, G20z.00, G20z.11, G21..00, G210.00, G210000, G210100, G211.00, G211000, G211100, G21z.00, G21z000, G21z011, G21z100, G21zz00, G22..00, G220.00, G221.00, G222.00, G22z.00, G22z.11, G23..00, G230.00, G231.00, G232.00, G233.00, G234.00, G23z.00, G2y..00, G2z..00, G672.00, G672.11, Gyu2.00, L122.00, L122000, L122100, L122300, L122z00, L127.00, L127z00, L128.00, L128000, L128200, TJC7.00, TJC7z00, U60C500, U60C511, U60C51A, 6146200, G24..00, G240.00, G240000, G240z00, G241.00, G241000, G241z00, G244.00, G24z.00, G24z000, G24z100, G24zz00, Gyu2100 |
| Ischemic Heart Disease | ICD10: I20, I21, I22, I23, I24, I25  Read codes: G3...00, G30..00, G300.00, G301.00, G301000, G30..11, G301100, G30..12, G30..13, G30..14, G30..15, G30..16, G30..17, G301z00, G302.00, G303.00, G304.00, G305.00, G306.00, G307.00, G307000, G307100, G308.00, G309.00, G30A.00, G30B.00, G30X.00, G30X00, G30y.00, G30y000, G30y100, G30y200, G30yz00, G30z.00, G31..00, G310.00, G310.11, G3...11, G311.00, G311000, G311011, G311100, G311.11, G311.12, G311.13, G311.14, G311200, G311300, G311400, G311500, G311z00, G3...12, G312.00, G3...13, G31y.00, G31y000, G31y100, G31y200, G31y300, G31yz00, G32..00, G32..11, G32..12, G33..00, G330.00, G330000, G330z00, G331.00, G331.11, G332.00, G33z.00, G33z000, G33z100, G33z200, G33z300, G33z400, G33z500, G33z600, G33z700, G33zz00, G34..00, G340.00, G340000, G340100, G340.11, G340.12, G342.00, G343.00, G344.00, G34y.00, G34y000, G34y100, G34yz00, G34z.00, G34z000, G35..00, G350.00, G351.00, G353.00, G35X.00, G36..00, G360.00, G361.00, G362.00, G363.00, G364.00, G365.00, G366.00, G38..00, G380.00, G381.00, G384.00, G38z.00, G3y..00, G3z..00 |
| Myocardial infarction | https://www.caliberresearch.org/portal/show/myo_infarct_gprdICD10: I21, I22, I23  Read codes: 14A3.00, 14A4.00, 14AH.00, G310.00, G32..00, G32..11, G32..12, G33z500, G30..11, G30..14, G30..17, G30A.00, G30X000, G307100, 323..00, 3233, 3234, 3235, 3236, 323Z.00, 889A.00, G30..00, G30..12, G30..13, G30..15, G30..16, G300.00, G301.00, G301000, G301100, G301z00, G302.00, G303.00, G304.00, G305.00, G306.00, G307.00, G307000, G308.00, G309.00, G30B.00, G30X.00, G30y.00, G30y000, G30y100, G30y200, G30yz00, G30z.00, G31y100, G38..00, G380.00, G381.00, G384.00, G38z.00, Gyu3400, G35..00, G350.00, G351.00, G353.00, G35X.00, G310.11, G36..00, G360.00, G361.00, G362.00, G363.00, G364.00, G365.00, G366.00, G501.00, TJC7z00, U60C500, U60C511, U60C51A, 6146200, G24..00, G240.00, G240000, G240z00, G241.00, G241000, G241z00, G244.00, G24z.00, G24z000, G24z100, G24zz00, Gyu2100 |
| Stroke | ICD10: I60, I61, I63, I64, I62.0, I62.1, I62.9, G46.3, G46.4, G46.5, G46.6, G46.7  Read codes: 14A7.00, 14A7.11, 14A7.12, 14AK.00, 1M4..00, 661M700, 661N700, 662e.00, 662e.11, 662M100, 662M200, 662M.00, 7P24200, 8HHM.00, 8IEC.00, 9h21.00, 9h22.00, 9h2..00, Fyu5600, G663.00, G664.00, G665.00, G666.00, G667.00, G668.00, G66..00, G66..11, G66..12, G66..13, G68X.00, Gyu6C00, L440.11, L440.12, ZV12511, ZV12512 |
| Heart failure | ICD10: I50  Read codes: 662f.00, 662F.00, 662g.00, 662G.00, 662h.00, 662H.00, 662i.00, G1yz100, G58..00, G580.00, G580000, G580100, G580.11, G580.12, G580.13, G580.14, G580200, G580300, G580400, G581.00, G581000, G58..11, G581.11, G581.12, G581.13, G582.00, G583.00, G583.11, G584.00, G58z.00, G58z.11, G58z.12 |
| Chronic obstructive pulmonary disease | ICD10: J40, J41, J42, J43, J44  Read codes: 14B3.12, H310000, H310.00, H310z00, H311000, H311100, H311.00, H311z00, H312000, H312011, H312100, H312200, H312300, H312.00, H312z00, H313.00, H31..00, H31y100, H31y.00, H31yz00, H31z.00, H320000, H320100, H320200, H320300, H320.00, H320z00, H321.00, H322.00, H32..00, H32y000, H32y100, H32y111, H32y200, H32y.00, H32yz00, H32z.00, H36..00, H37..00, H38..00, H39..00, H3A..00, H3...00, H3...11, H3y0.00, H3y1.00, H3y..00, H3y..11, H3z..00, H3z..11, H464000, H464100, H583200, Hyu3000, Hyu3100 |
| Chronic kidney disease | ICD10: N00, N10, N17, N01, N03, N052, N053, N054, N055, N056, N072, N073, N074, N181, N182, N183, N184, N185, N189, N19, N25, Y841, Z49, Z992, T861, Z940  Read codes: 1Z16.00, 1Z1B.00, 1Z1C.00, 1Z1D.00, 1Z1E.00, 1Z1F.00, 1Z1G.00, 1Z1H.00, 1Z1J.00, 1Z1K.00, 1Z1L.00, 7B06300, 8L50.00, K01..00, K010.00, K011.00, K012.00, K013.00, K013.11, K013.12, K014.00, K015.00, K016.00, K017.00, K018.00, K019.00, K01A.00, K01B.00, K01w.00, K01w000, K01x000, K01x100, K01x111, K01x200, K01x300, K01x400, K01x411, K01y.00, K01z.00, K02..00, K020.00, K021.00, K02..11, K02..12, K022.00, K023.00, K02y.00, K02y000, K02y200, K02y300, K02yz00, K02z.00, K05..00, K050.00, K05..11, K05..12, K0D..00, K100.00, K100000, K100100, K100200, K100300, K100400, K100500, K100600, K100z00, SP08300, TB00100, TB00111, ZV42000, 1Z12.00, 1Z13.00, 1Z14.00, 1Z15.00 |
| Cancer | ICD10: C00 to C97  Read codes: B0...00, B00..00, B000.00, B000000, B000100, B000z00, B001.00, B001000, B00..11, B001100, B001z00, B002.00, B002100, B002200, B002300, B002z00, B003.00, B003000, B003100, B003200, B003300, B003z00, B004.00, B004000, B004200, B004300, B005.00, B006.00, B007.00, B00z000, B00z100, B00zz00, B01..00, B010.00, B010000, B010.11, B010z00, B0...11, B011.00, B011z00, B012.00, B013.00, B013000, B013100, B013z00, B014.00, B015.00, B016.00, B017.00, B01y.00, B01z.00, B02..00, B020.00, B021.00, B022.00, B02y.00, B02z.00, B03..00, B030.00, B031.00, B03y.00, B03z.00, B04..00, B040.00, B041.00, B042.00, B04y.00, B04z.00, B05..00, B050.00, B050.11, B051.00, B051000, B051100, B052.00, B053.00, B054.00, B055.00, B055000, B055100, B055z00, B056.00, B057.00, B05y.00, B05z.00, B05z000, B06..00, B060.00, B060000, B060100, B060200, B060z00, B061.00, B062.00, B062000, B062100, B062200, B062300, B062z00, B063.00, B064.00, B064000, B064100, B064z00, B065.00, B066.00, B067.00, B06y.00, B06yz00, B06z.00, B07..00, B070.00, B071.00, B071000, B071100, B071z00, B072.00, B072000, B072z00, B073.00, B073200, B073z00, B074.00, B07y.00, B07z.00, B08..00, B080.00, B081.00, B082.00, B083.00, B08y.00, B08z.00, B0z..00, B0z0.00, B0z1.00, B0z2.00, B0zy.00, B0zz.00, B1...00, B10..00, B100.00, B101.00, B102.00, B103.00, B104.00, B105.00, B106.00, B107.00, B10y.00, B10z.00, B10z.11, B11..00, B110.00, B110000, B110100, B110111, B110z00, B1...11, B111.00, B111000, B11..11, B111100, B111z00, B112.00, B113.00, B114.00, B115.00, B116.00, B117.00, B118.00, B119.00, B11y.00, B11y000, B11y100, B11yz00, B11z.00, B12..00, B120.00, B121.00, B122.00, B123.00, B124.00, B12y.00, B12z.00, B13..00, B130.00, B131.00, B132.00, B133.00, B134.00, B134.11, B135.00, B136.00, B137.00, B138.00, B139.00, B13y.00, B13z.00, B13z.11, B14..00, B140.00, B141.00, B141.11, B141.12, B142.00, B142000, B142.11, B143.00, B14y.00, B14z.00, B15..00, B150.00, B150000, B150100, B150200, B150300, B150z00, B151.00, B151000, B151200, B151400, B151z00, B152.00, B153.00, B15z.00, B16..00, B160.00, B160.11, B161.00, B161000, B161100, B161200, B161211, B161z00, B162.00, B163.00, B16y.00, B16z.00, B17..00, B170.00, B171.00, B172.00, B173.00, B174.00, B175.00, B17y.00, B17y000, B17yz00, B17z.00, B18..00, B180.00, B180100, B180200, B180z00, B181.00, B182.00, B18y.00, B18y100, B18y200, B18y300, B18y400, B18y500, B18y600, B18y700, B18yz00, B18z.00, B1z..00, B1z0.00, B1z0.11, B1z1.00, B1z1100, B1z1z00, B1z2.00, B1zy.00, B1zz.00, B2...00, B20..00, B200.00, B200000, B200100, B200200, B200300, B200z00, B201.00, B201100, B201200, B201300, B201z00, B202.00, B203.00, B204.00, B205.00, B206.00, B20y.00, B20z.00, B21..00, B210.00, B2...11, B211.00, B212.00, B213.00, B213000, B213100, B213300, B213z00, B214.00, B215.00, B21y.00, B21z.00, B22..00, B220.00, B220100, B220z00, B221.00, B221000, B221100, B221z00, B222.00, B222000, B222100, B222.11, B222z00, B223.00, B223000, B223100, B223z00, B224.00, B224000, B224100, B224z00, B225.00, B226.00, B22y.00, B22z.00, B22z.11, B23..00, B230.00, B232.00, B23y.00, B23z.00, B24..00, B240.00, B241.00, B241000, B241200, B241300, B241400, B241z00, B242.00, B243.00, B24X.00, B24y.00, B24z.00, B25..00, B26..00, B2z..00, B2z0.00, B2zy.00, B2zz.00, B3...00, B30..00, B300.00, B300000, B300100, B300200, B300300, B300400, B300500, B300600, B300700, B300800, B300900, B300A00, B300B00, B300C00, B300z00, B301.00, B30..11, B30..12, B302.00, B302000, B302100, B302200, B302z00, B303.00, B303000, B303100, B303200, B303300, B303400, B303500, B303z00, B304.00, B304000, B304200, B304300, B304400, B304z00, B305.00, B305000, B305100, B305.12, B305C00, B305D00, B305z00, B306.00, B306000, B306100, B306200, B306300, B306400, B306500, B306z00, B307.00, B307000, B307100, B307200, B307300, B307z00, B308.00, B308100, B308200, B308300, B308400, B308500, B308600, B308700, B308800, B308z00 |
| Valvular heart disease | ICD10: I05, I06, I07, I08, I34, I35, I36, I37  Read codes: 7911300, G121.00, G121.12, G122.00, G541011, G541012, G541100, G541211. G541212, G541400, G541500, P63..00, P64..00, P640.00, P64z.00, G111.00, G111.12, G112.00, G112.13, G113.00, G131.00, G131.14, G132.00, G132.13, G133.11, G133.12, G540.12, G540.14, G540.16, P66..00, G141100, G141200, G543011, G543012, G543213, G543215, G543400, H585.00, H585200, G140100, G140111, G140200, G14021X, G140400, G140413, G140500, G140514, G542011, G542012, G542200, G54z500 |
| Septic shock | ICD10: R65.2  Read codes: 1JX0., 1JX1., 1JX2 |

Note: Codes available from HDRUK CALIBER Github repository [1, 2] and derived from Kuan and colleagues [3].

**References:**

1. HDR UK. HDR UK National Phenotype Library. GitHub repository. 2021. Available at: <https://github.com/spiros/hdr-caliber-phenotype-library> , last accessed 18^th^ January 2025

2. HDR UK. HDR UK National Phenotype Library. Phenotype Library website. 2021. Available at:

<https://phenotypes.healthdatagateway.org/phenotypes/?collection_ids=21> , last accessed 18^th^ January 2025

3. Kuan V, Denaxas S, Gonzalez-Izquierdo A, et al. A chronological map of 308 physical and mental health conditions from 4 million individuals in the English National Health Service. Lancet Digit. Health. 2019;1:e63–e77. doi: 10.1016/S2589-7500(19)30012-3.

**Supplemental Table 2.** Study population and baselines after propensity score matching

| **Variable** | **Comparison a**  **New-onset AF in sepsis vs. Sepsis without New-onset AF** | | | **Comparison b**  **New-onset AF in sepsis vs. New-onset AF without sepsis** | | |
| --- | --- | --- | --- | --- | --- | --- |
|  | Sepsis with new-onset AF)  (n=7,691) | Sepsis without new-onset AF)  (n=7,691) | Standardized Mean Difference | Sepsis with new-onset AF)  (n=7,529) | New-onset AF without sepsis)  (n=7,529) | Standardized Mean Difference |
| Age > 70 | 6,043 (78.57%) | 6,127 (79.66%) | 0.020 | 5,672 (75.34%) | 5,844 (77.62%) | 0.005 |
| Female Sex | 3,640 (47.33%) | 3,653 (47.50%) | 0.001 | 3,565 (47.35%) | 3,482 (46.25%) | 0.003 |
| Non-White Ethnicity | 181 (2.35%) | 161 (2.09%) | 0.004 | 183 (2.43%) | 155 (2.06%) | 0.017 |
| IMD  1 (most deprived)  2^nd^  3^rd^  4^th^  5 (least deprived) | 1,280 (16.64%)  1,577 (20.50%)  1,416 (18.41%)  1,613 (20.97%)  1,805 (23.47%) | 1,315 (17.10%)  1,582 (20.57%)  1,358 (17.66%)  1,630 (21.19%)  1,806 (23.48%) | 0.001  0.010  0.007  0.004  0.007 | 1,256 (16.68%)  1,554 (20.64%)  1,378 (18.30%)  1,579 (20.97%)  1,762 (23.40%) | 1,226 (16.28%)  1,626 (21.60%)  1,342 (17.82%)  1,589 (21.11%)  1,746 (23.19%) | 0.001  0.001  <0.001  0.002  <0.001 |
| Smokers | 6,351 (82.58%) | 6,317 (82.13%) | 0.012 | 6,139 (81.54%) | 6,236 (82.83%) | 0.007 |
| CKD | 2,199 (28.59%) | 2,104 (27.36%) | 0.004 | 1,629 (21.64%) | 1,581 (21.00%) | 0.004 |
| COPD | 4,337 (56.39%) | 4,357 (56.65%) | 0.008 | 3,876 (51.48%) | 3,876 (51.48%) | <0.001 |
| Diabetes Mellitus | 2,181 (28.36%) | 2,194 (28.53%) | 0.001 | 1,936 (25.71%) | 1,936 (25.71%) | 0.002 |
| Heart Failure | 2,881 (37.46%) | 2,886 (37.52%) | 0.005 | 2,092 (27.79%) | 2,028 (26.94%) | 0.001 |
| Hypertension | 5,833 (75.84%) | 5,858 (76.17%) | 0.027 | 5,340 (70.93%) | 5,429 (72.11%) | 0.002 |
| Ischemic Heart Disease | 152 (1.98%) | 122 (1.59%) | 0.043 | 103 (1.37%) | 88 (1.17%) | 0.001 |
| Valvular heart disease | 766 (9.96%) | 667 (8.67%) | 0.028 | 601 (7.98%) | 459 (6.10%) | 0.019 |

Legend: AF: Atrial Fibrillation; IMD: Index of Multiple Deprivation; CKD: Chronic Kidney Disease; COPD: Chronic Obstructive Pulmonary Disease.

**Supplemental Table 3.** Mortality and cardiovascular outcomes after propensity score matching

| **Comparison a**  **New-onset AF in sepsis vs. Sepsis without New-onset AF** | | **Comparison b**  **New-onset AF in sepsis vs. New-onset AF without sepsis** | |
| --- | --- | --- | --- |
| In-hospital mortality in 7-days | | | |
| Odds Ratio (95% CI) | P Value | Odds Ratio (95% CI) | P Value |
| 2.12 (1.91, 2.36) | <0.001 | 1.40 (1.23, 1.61) | <0.001 |
| In-hospital mortality in 30-days | | | |
| Odds Ratio (95% CI) | P Value | Odds Ratio (95% CI) | P Value |
| 2.58 (2.38, 2.80) | <0.001 | 1.36 (1.25, 1.49) | <0.001 |
| Mortality post-discharge | | | |
| Hazard Ratio (95% CI) | P Value | Hazard Ratio (95% CI) | P Value |
| 1.06 (1.01, 1.11) | 0.024 | 1.40 (1.35, 1.45) | <0.001 |
| Stroke post-discharge | | | |
| Hazard Ratio (95% CI) | P Value | Hazard Ratio (95% CI) | P Value |
| 1.16 (1.04, 1.30) | 0.008 | 1.04 (0.95, 1.13) | 0.445 |
| Heart failure hospitalisation post-discharge | | | |
| Hazard Ratio (95% CI) | P Value | Hazard Ratio (95% CI) | P Value |
| 1.74 (1.59, 1.91) | <0.001 | 1.40 (1.31, 1.50) | <0.001 |
| Myocardial infarction hospitalisation post-discharge | | | |
| Hazard Ratio (95% CI) | P Value | Hazard Ratio (95% CI) | P Value |
| 1.29 (1.16, 1.45) | 0.002 | 1.17 (1.07, 1.28) | <0.001 |

**Supplemental Table 4.** In-hospital septic shock in new-onset AF with sepsis vs. sepsis without new-onset AF after propensity score matching

| In-hospital septic shock in 7-days | |
| --- | --- |
| Odds Ratio (95% CI) | P Value |
| 1.61 (1.15, 2.27) | 0.006 |
| In-hospital septic shock in 30-days | |
| Odds Ratio (95% CI) | P Value |
| 1.63 (1.16, 2.29) | 0.005 |
